# Potential Starter *Candida*te Based on Safety and Technological Evaluation of *Lactococcus lactis* from Kimchi, Korean Traditional Fermented Vegetables

**DOI:** 10.4014/jmb.2501.01015

**Published:** 2025-04-24

**Authors:** Yura Moon, Sojeong Heo, Gawon Lee, Sumin Lee, Minkyeong Kim, Moon-Hee Sung, Do-Won Jeong

**Affiliations:** 1 Department of Food and Nutrition, Dongduk Women’s University, Seoul 02748, Republic of Korea; 2 KookminBio Corporation, Seoul 02826, Republic of Korea

**Keywords:** *Lactococcus lactis*, starter culture, safety assessment, technological property, antibacterial activity

## Abstract

Continuous screening of suitable starter candidates for fermented foods necessary based on safety and technological evaluations. For this purpose, 34 *Lactococcus lactis* strains isolated from kimchi were evaluated to select potential starter candidates. All tested strains were susceptible to ampicillin, chloramphenicol, clindamycin, erythromycin, gentamicin, kanamycin, and vancomycin. However, 32 strains exhibited resistance to streptomycin, while 2 showed resistance to tetracycline. None of the strains demonstrated hemolytic activity. All strains exhibited protease activity and acid production, but none displayed amylase or lipase activity. Notably, all strains exhibited antibacterial activity against *Listeria monocytogenes* ATCC 19111 and *Salmonella enterica* KCCM 11862. Furthermore, antibacterial activity was observed in 70.6%, 79.4%, 79.4%, 73.5%, and 32.4% of strains against *Bacillus cereus* KCCM 11341, *Enterococcus faecalis* KCTC 2011, *Staphylococcus aureus* ATCC 12692, *Flavobacterium* sp. KCCM 11374, and *Vibrio parahaemolyticus* KCTC 2729, respectively. Based on these assessments, *L. lactis* strain DMLL15 was identified as the most promising starter candidate. This strain was sensitive to nine antibiotics, exhibited non-hemolytic activity, demonstrated strong protease activity and acid production, and displayed antibacterial activity against seven pathogenic and spoilage bacteria. These findings support the safety assessment results and highlight the potential application of *L. lactis* DMLL15 as a starter candidate in the food industry.

## Introduction

Food spoilage occurring between processing and consumption can significantly undermine consumer trust in the product. For food processors, this results in severe economic losses from restoring consumer confidence, disposing of spoiled products, and reorganizing processing procedures [1]. To prevent spoilage during processing, preservatives such as potassium sorbate are frequently added. However, due to negative consumer perceptions of food additives, there is an increasing trend toward natural antimicrobial substances as alternatives. Currently, nisin is the only approved natural antimicrobial substance; thus, biopreservation methods involving microorganisms that produce antimicrobial compounds are sometimes utilized [2]. Consequently, screening for strains with antimicrobial activity has been ongoing for an extended period [3].

*Lactococcus lactis* is a homofermentative lactic acid bacterium that produces two molecules of lactic acid from glucose [4]. Additionally, it generates byproducts such as diacetyl, hydrogen peroxide, acetaldehyde, and bacteriocins [4, 5]. These byproducts demonstrate antimicrobial and antifungal properties, contributing to the preservation of fermented foods, enhancing their sensory attributes, and providing nutritional and health advantages [6-8]. Consequently, employing *L. lactis* as a starter culture can suppress the growth of spoilage and pathogenic bacteria due to the low pH from lactic acid and the production of antimicrobial substances, thus reducing the risk of spoilage or foodborne illness.

*L. lactis* is commonly found in fermented dairy products such as cheese and cultured butter, and has long been utilized as a starter culture in dairy fermentation [9, 10]. However, it is seldom isolated from fermented vegetable products. In a previous study, our research group isolated and identified 1,219 strains from kimchi containing seafood to explore a variety of starter cultures that could enhance the sensory characteristics of kimchi and accommodate individual preferences in fermented foods [11-13]. Among these, *L. lactis* comprised 35 strains (2.8%). Although *L. lactis* is not a dominant strain in kimchi, its numerous benefits necessitate the selection of safe, high-performing, and health-promoting strains from this species. Thus, this study endeavors to select candidate starter strains by assessing the safety and technological properties, including antimicrobial activity among 35 *L. lactis* strains isolated from kimchi. Should this study successfully isolate and secure safe and advanced strains of *L. lactis* with antimicrobial properties, they may serve as starter cultures for both kimchi fermentation and the dairy industry, where their antimicrobial substances could be utilized in food processing.

## Materials and Methods

### Bacterial Strains and Culture Conditions

In this experiment, 34 strains were used, excluding one strain that was reanalyzed and found not to be *L. lactis* among the 35 *L. lactis* strains isolated from kimchi in a previous experiment [11-13]. Type strain *L. lactis* KACC 13877^T^ was employed as a control. *L. lactis* was cultured in MRS broth (MB Cell, Republic of Korea) at 30°C for 18 h to preserve bacterial traits.

### Antibiotic Resistance Tests

The antibiotic resistance experiment was conducted in two stages. First, antibiotic susceptibility was assessed using agar containing antibiotics. Then, for antibiotics that showed resistance, additional confirmation was performed using the Minimum Inhibitory Concentration (MIC) tests.

For agar analysis, nine antibiotics and their respective cut-off values, as suggested by the European Food Safety Authority (EFSA) antibiotic susceptibility guidelines, were used: 2 μg/ml ampicillin, 8 μg/ml chloramphenicol, 1 μg/ml clindamycin, 1 μg/ml erythromycin, 32 μg/ml gentamicin, 64 μg/ml kanamycin, 32 μg/ml streptomycin, 4 μg/ml tetracycline, and 4 μg/ml vancomycin [14]. The strains were inoculated onto Brain Heart Infusion (BHI; Becton, Dickinson and Co., USA) agar containing each antibiotic, a medium applied to *Enterococcus* among lactic acid bacteria according to the Clinical and Laboratory Standards Institute guidelines [15], using the tooth-picking method, and incubated at 30°C for 24 h. Strains that exhibited growth on the medium were considered resistant.

The MIC tests were conducted on two antibiotics that exhibited resistance (streptomycin and tetracycline). The MICs were determined using the broth microdilution method [14]. Streptomycin and tetracycline were prepared in serial twofold working dilutions in deionized water, with final concentrations in each 96-microwell plate ranging from 0.5 to 256 mg/l. Bacterial strains were cultured twice in MRS broth and adjusted to a 0.5 McFarland turbidity standard. Subsequently, each suspension was diluted 1:100 in BHI broth to attain the proper inoculum concentration. The final inoculum density reached 5 × 10^5^ colony-forming units/ml, and 200 μl was transferred to each well of the 96-microwell plate. The MIC of streptomycin and tetracycline was identified as the lowest concentration at which no growth was observed in the wells following incubation at 30°C for 18 h. The MIC results were verified through at least three independently performed tests. All experiments were conducted at least three times on separate days. Strains with the MICs exceeding the breakpoint are considered resistant [15].

### PCR for the Identification of Antibiotic Resistance

Genomic DNA from *L. lactis* strains was extracted using a Genomic DNA Prep Kit (Nanohelix, Republic of Korea). The amplification of resistance genes for streptomycin and tetracycline was conducted with specific primer sets as indicated in Table 1 using a T-3000 Thermocycler (Biometra, Germany) (Table 1). The PCR mixture contained 30 ng of template DNA, 0.5 μM of each primer, 1.25 units of *Taq* polymerase (Inclone Biotech Co., Republic of Korea), 10 mM dNTPs, and 2.5 mM MgCl_2_. The samples were initially heated at 94°C for 3 min, followed by 30 cycles consisting of 1 min at 94°C, 1 min or 30 sec at 50 -57°C, and 1 min or 30 sec at 72°C. PCR - amplified fragments were identified on 1.5% agarose gel.

### Hemolytic Activity Test

Tryptic Soy Agar (TSA; Becton, Dickinson and Co.) was supplemented with either 5% (v/v) rabbit blood (MB Cell) or 5% (v/v) sheep blood (MB Cell) for testing α- or β-hemolytic activity, respectively. α-hemolytic activity was detected after incubation at 30°C for 24 h, and β-hemolytic activity was determined by a cold shock at 4°C after a 24 h incubation at 30°C. Hemolytic activities were identified by observing clear lytic zones around the colonies on each blood-containing TSA plate. *Staphylococcus aureus* USA300-p23 and RN4220 served as positive and negative controls, respectively, in these analyses. All experiments were repeated at least three times on separate days.

### Acid Production and Enzyme Activity

Acid production was determined using TSA with 0.5% (w/v) glucose and 0.7% (w/v) CaCO_3_. Protease activity was assessed on TSA containing 0.5% (w/v) glucose and 2% (w/v) skim milk. Amylase activity was evaluated using TSA with 0.5% (w/v) glucose and 1% (w/v) starch. Lipase activity was tested on tributyrin agar (Sigma-Aldrich, USA) containing 1% (v/v) tributyrin and 0.5% (w/v) glucose. The tributyrin-supplemented medium was emulsified by sonication prior to autoclaving. To evaluate enzyme activity other than amylase, filter paper discs were placed on the surface of each substrate-supplemented agar medium and 10 μl of *L. lactis* cultured in MRS broth was applied to these discs. The plates were then incubated at 30°C for 24 h. The relative size of the clearing zone around the filter paper disc served as an indicator of enzyme activity. The effect of NaCl on protease activity was assessed by adding NaCl to each medium to a final concentration of 6.5% (w/v). To evaluate amylase activity, 5μl of *L. lactis* cultured in MRS broth was applied to the substrate-supplemented agar medium surface and incubated at 30°C for 24 h. Following incubation, the plate was left at room temperature for an additional 24 h, after which a 1% iodine solution was applied. The relative size of the clearing zone around the culture served as an indicator of amylase activity. All experiments were conducted at least twice on separate days.

### Antibacterial Activity

The antibacterial activities of *L. lactis* against nine foodborne pathogenic bacteria (*Bacillus cereus* KCCM 11341, *Enterococcus faecalis* KCTC 2011, *Listeria monocytogenes* ATCC 19111, *S. aureus* ATCC 12692, *Alcaligenes xylosoxidans* KCCM 40240, *Escherichia coli* O157:H7 EDL 933, *Flavobacterium* sp. KCCM 11374, *Salmonella enterica* KCCM 11862 and *Vibrio parahaemolyticus* KCTC 2729) were assessed using the agar well diffusion method. Overnight cultures of pathogens in TSB (Becton, Dickinson and Co.) were inoculated at 1% (v/v) into fresh TSB and incubated until an OD_600_ of 1.0 was reached; 200 μl of each culture was then introduced onto TSA plates. A 6 mm diameter hole was aseptically punched using a sterile cork borer, and 50 μl of concentrated *L. lactis* supernatant was introduced into the well. These agar plates were then incubated at 30°C for 24 h. The concentrated supernatant of *L. lactis* was prepared by incubating the culture for 24 h at 30°C in MRS broth, followed by centrifugation and five-fold concentration using a HyperVAC (Centrifugal Vacuum Concentrator VC2124, Hanil Scientific Inc., Republic of Korea). The size of the clearing zone around the punched hole served as an indicator of antibacterial efficacy. All experiments were repeated at least twice on separate days.

### Statistical Analysis

Duncan’s multiple range test following a one-way analysis of variance (ANOVA) was used to evaluate significant differences between average values of acid production, enzyme activities and antibacterial activities. Values with *p* < 0.05 were considered statistically significant. All statistical analysis was per-formed using the SPSS software package (version 27.0; SPSS, IBM, USA).

## Results and Discussion

### Safety Properties of *L. lactis* Strain Isolated from Kimchi

EFSA has provided guidelines for evaluating the antibiotic resistance of bacteria intended for use in food and feed, particularly with regard to their impact on human and animal health [15]. These guidelines aim to assess the potential transfer of antibiotic resistance genes from antibiotic-resistant bacteria to humans or animals. The resistance criteria are based on cut-off values for each antibiotic, established by the European Committee on Antimicrobial Susceptibility Testing (EUCAST, http://www.eucast.org/) through monitoring data. A total of nine antibiotics were evaluated for *L. lactis*: ampicillin, chloramphenicol, clindamycin, erythromycin, gentamicin, kanamycin, streptomycin, tetracycline, and vancomycin. Resistance was assessed by determining the ability of the strains to grow in media containing the cut-off value concentrations of each antibiotic as specified in the EFSA guidelines. None of the tested strains grew on media containing these seven antibiotics: ampicillin, chloramphenicol, clindamycin, erythromycin, gentamicin, kanamycin, and vancomycin. However, 32 strains (94.1%) were able to grow in the presence of streptomycin at the cut-off concentration (32 mg/l), and 2 strains (5.9%) showed growth in the presence of tetracycline at the cut-off concentration (4 mg/l).

**Fig. 1:**
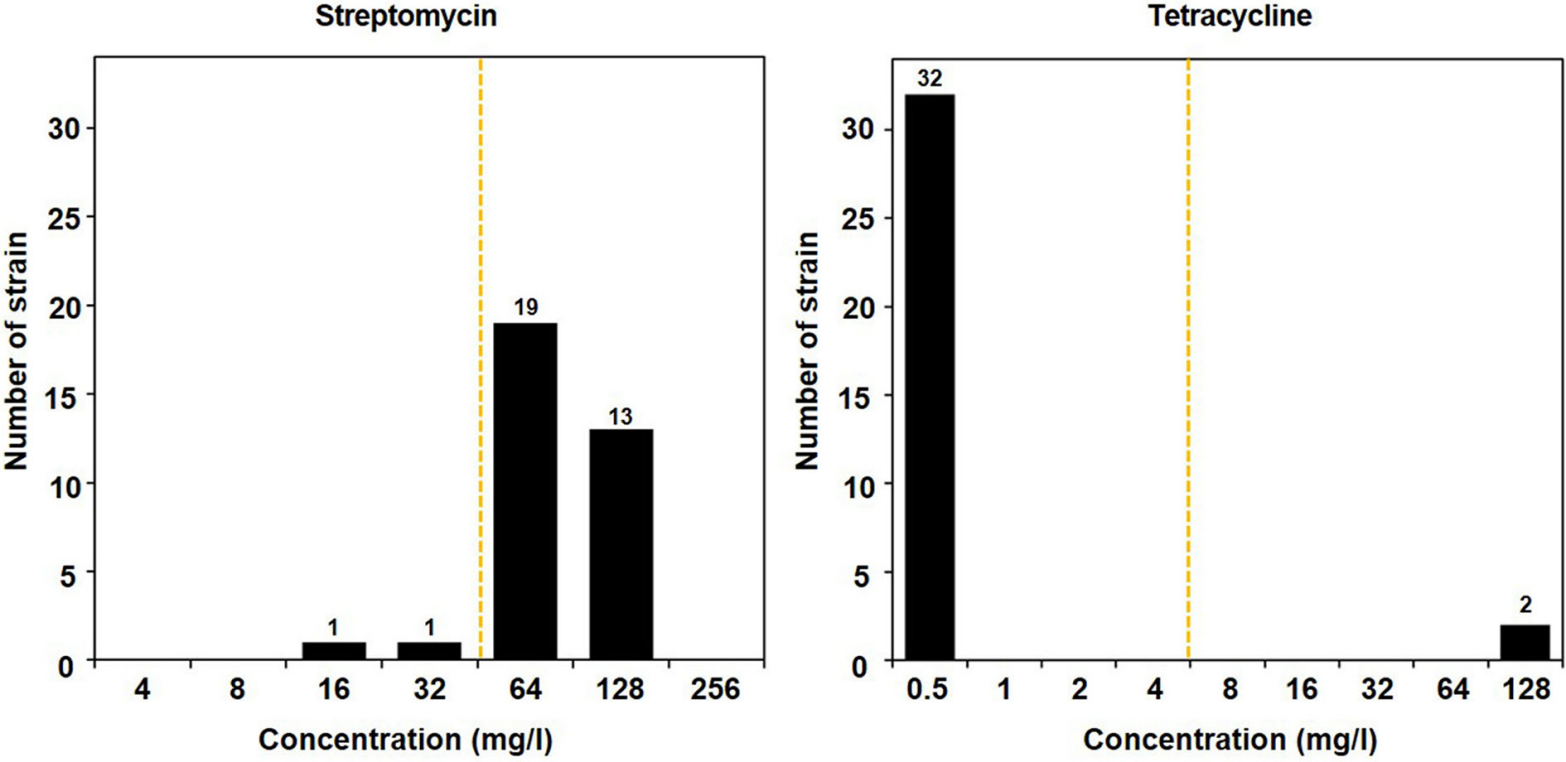
Minimal inhibitory concentration (mg/l) against streptomycin and tetracycline of *L. lactis* 34 strains. The vertical yellow dotted line represents the breakpoint for *L. lactis* as defined by EFSA [15]. The numbers on the bars represent the number of strains for which the given antibiotic concentration is the MIC.

The MIC for two antibiotics, streptomycin and tetracycline, which showed growth at the cut-off value concentration among the nine tested antibiotics, was analyzed. The MICs of streptomycin and tetracycline for the tested 34 strains are summarized in Fig. 1. For streptomycin, 32 strains demonstrated higher resistance than the EFSA-specified breakpoint of 32 mg/l, with MIC values ranging from 16 to 128 mg/l for the 34 strains (Fig. 1). These results are consistent with the outcomes observed in media containing the cut-off value concentration. According to a recent study, the MIC values for strains range from 0.5 to 64 mg/l [19, 20]. This also showed that some strains exhibited MIC values higher than the breakpoint, leading to speculation that the EFSA’s breakpoint value for *L. lactis* may be set too low. Although the MIC values for streptomycin were elevated in our experiment, the normal distribution pattern of the MICs suggested that the resistance is likely a species-specific characteristic or intrinsic resistance rather than an acquired genetic trait. To verify this, the presence of streptomycin resistance genes, *strA* (streptomycin resistance protein A), *strB* (streptomycin resistance protein B), *aadA* (aminoglycoside adenylyltransferase A), and *aadE* (amino glycoside adenylyltransferase E), was examined using PCR. None of the 34 strains showed amplification for any of these genes, indicating that the *L. lactis* strains did not possess the acquired resistance genes *strA*, *strB*, *aadA*, or *aadE* for streptomycin. Given the high resistance observed and the lack of amplification of these genes, the resistance to streptomycin is likely intrinsic. Similarly, the two strains that exhibited resistance to tetracycline were tested for the presence of tetracycline resistance genes *tetK* (tetracycline resistance protein K) and *tetM* (tetracycline resistance protein M) using PCR, but neither gene was amplified. This suggests that the resistance in these two strains is also likely intrinsic rather than acquired. As various factors contribute to antibiotic resistance, further research is essential to identify the specific genes involved in tetracycline resistance.

EFSA evaluates antibiotic resistance for all bacteria in food and feed, but assesses toxicity, including hemolytic activity, only for specific species [21]. The aim of toxicity assessment is to precisely determine toxic factors and ascertain their absence in specific species [22]. For instance, bacteria from the *Bacillus* genus, including the pathogenic *B. cereus*, are screened for toxic factors. A common method used for this purpose is observing hemolytic activity. Although *L. lactis* does not require verification of toxic factors including hemolysis, a safety test using blood agar was conducted. As anticipated, none of the strains showed either α-hemolysis or β-hemolysis.

### Technological Properties of *L. lactis*

The studies on antibiotic resistance and hemolytic activity relate to the safety of *L. lactis*. While safety is essential for starter culture candidates, technological characteristics such as enzyme activity are critical for a starter culturés performance. Amylase, protease, and lipase activities enhance the sensory qualities of fermented foods by generating taste and aroma compounds from the protein and lipid constituents of food [23-27]. Acids in fermented foods improve both flavor and preservation [28]. Given the significant role of enzyme activity in enhancing the sensory attributes of fermented foods through the production of taste and aroma compounds, we evaluated the amylase, protease, and lipase activities, as well as acid production, in our *L. lactis* strains.

All strains tested in the experiment demonstrated protease activity in an environment devoid of added salt (Table 2). Even when the salt concentration in the medium was increased to 3.5%, the strains formed zones, although the zone size was diminished. Among these, 9 strains (26.5%) formed a clear zone of 1.05 ± 0.07 or larger, indicating relatively high protease activity. Among them, strain DMLL15 exhibited the highest activity. Moreover, all tested strains produced acid (Table 2). However, none of the strains exhibited amylase or lipase activity.

Fermented foods, including dairy products where *L. lactis* is predominantly found, typically do not contain high salt concentrations [29]. The food from which *L. lactis* was isolated in this study is kimchi, which typically has a salt concentration ranging from 2 5% [11, 30, 31]. Therefore, if these strains are to be used as starter cultures for kimchi, those exhibiting superior enzyme activity at this salt concentration could notably enhance the sensory qualities of the fermented product. In particular, protease activity was observed at a 3.5% salt concentration, suggesting that it may contribute to the breakdown of proteins in raw materials, thereby generating flavor and aroma compounds, and previous studies have highlighted the importance of salt-tolerant enzymes in flavor development within high-salt fermented foods [24, 25]. However, since no lipase or amylase activity was detected, it is expected that there will be no sensory characteristics derived from the breakdown of fats and carbohydrates. Consequently, we suggest that strains demonstrating high protease activity at a salt concentration of 3.5% exhibit great potential as starter culture candidates.

### Antibacterial Activity of *L. lactis*

**Fig. 2:**
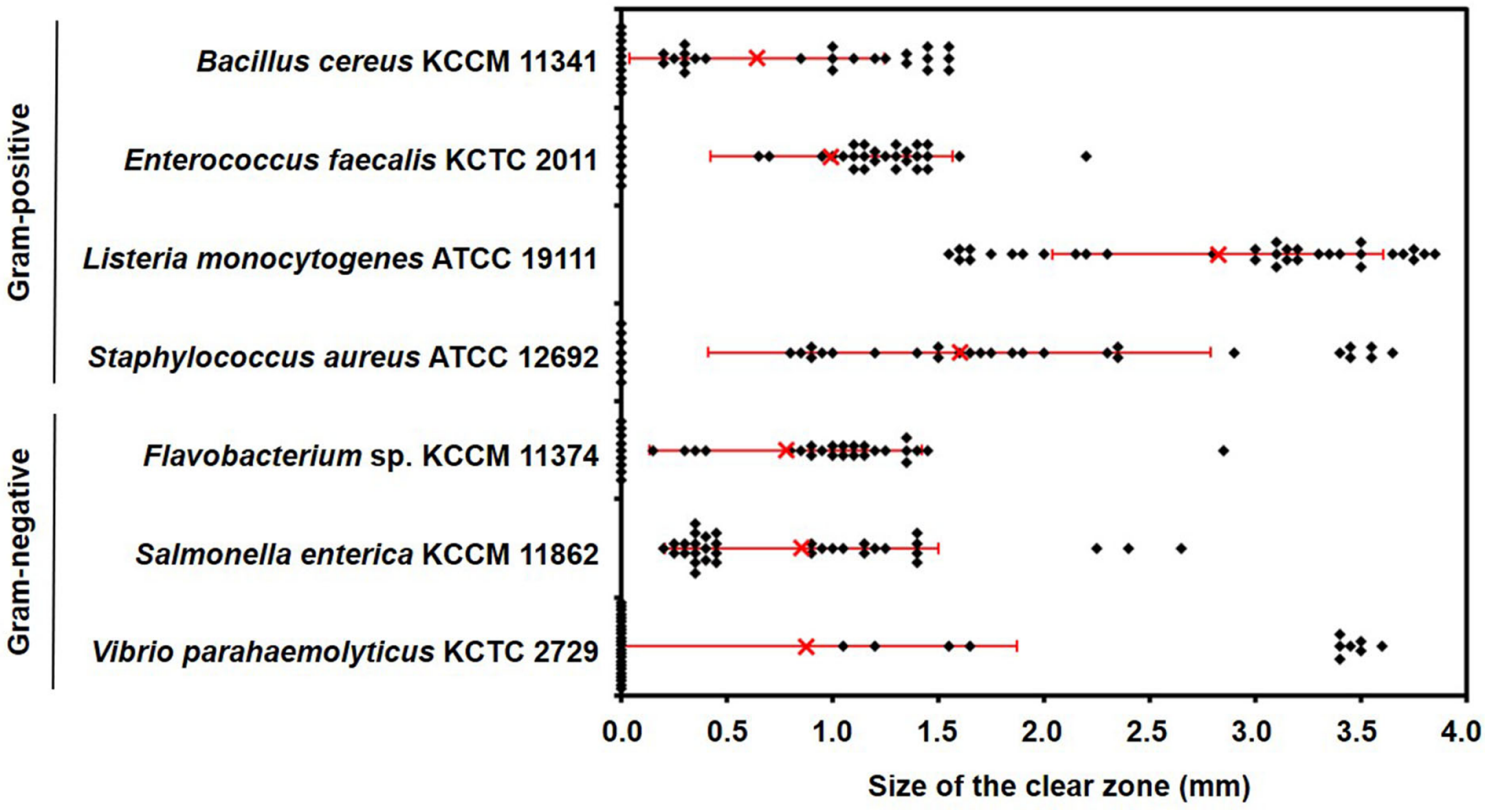
Antibacterial activity of *L. lactis* 34 strains against food pathogens using the agar well diffusion method. The formation of a clear zone around the well was used as an indicator of antibacterial activity. Each dot represents one strain. The red 'X' was indicated the mean, while the line represented the standard deviation.

Strains that are safe, exhibit strong enzyme activity, and possess antibacterial properties meet the criteria for use as starter cultures in fermented foods [32]. Thus, the antibacterial activities of *L. lactis* strains were assessed using the agar well diffusion method. The indicator strains for assessing antibacterial activity included four Gram-positive bacteria (*B. cereus* KCCM 11341, *E. faecalis* KCTC 2011, *L. monocytogenes* ATCC 19111, and *S. aureus* ATCC 12692) and five Gram-negative bacteria (*A. xylosoxidans* KCCM 40240, *E. coli* O157:H7 EDL 933, *Flavobacterium* sp. KCCM 11374, *S. enterica* KCCM 11862, and *V. parahaemolyticus* KCTC 2729) (Fig. 2). All *L. lactis* strains exhibited no antibacterial activity against *A. xylosoxidans* KCCM 40240 and *E. coli* O157:H7 EDL 933. Conversely, all strains displayed antibacterial activity against *L. monocytogenes* ATCC 19111 and *S. enterica* KCCM 11862, though the size of the inhibition zones varied. For the other five strains (*B. cereus* KCCM 11341, *E. faecalis* KCTC 2011, *S. aureus* ATCC 12692, *Flavobacterium* sp. KCCM 11374, and *V. parahaemolyticus* KCTC 2729), 70.6%, 79.4%, 79.3%, 73.5%, and 32.3% of the *L. lactis* strains, respectively, formed inhibition zones (Fig. 2), demonstrating strain-specific antibacterial activity. Notably, the strains demonstrated the highest antibacterial activity against *L. monocytogenes* ATCC 19111, *S. aureus* ATCC 12692, and *V. parahaemolyticus* KCTC 2729, as evidenced by the size of the inhibition zones. Most importantly, 11 out of the 34 strains showed antibacterial activity against seven food spoilage or foodborne pathogens, even if the activity was weak. Among them, strain DMLL15 distinctly formed inhibition zones against all seven pathogens, exhibiting the highest antibacterial activity. These findings suggest that utilizing strains with antibacterial activity as starter cultures in fermented foods could inhibit the growth of targeted harmful bacteria.

### Selection of Strain DMLL15 as a Starter *Candida*te

**Fig. 3:**
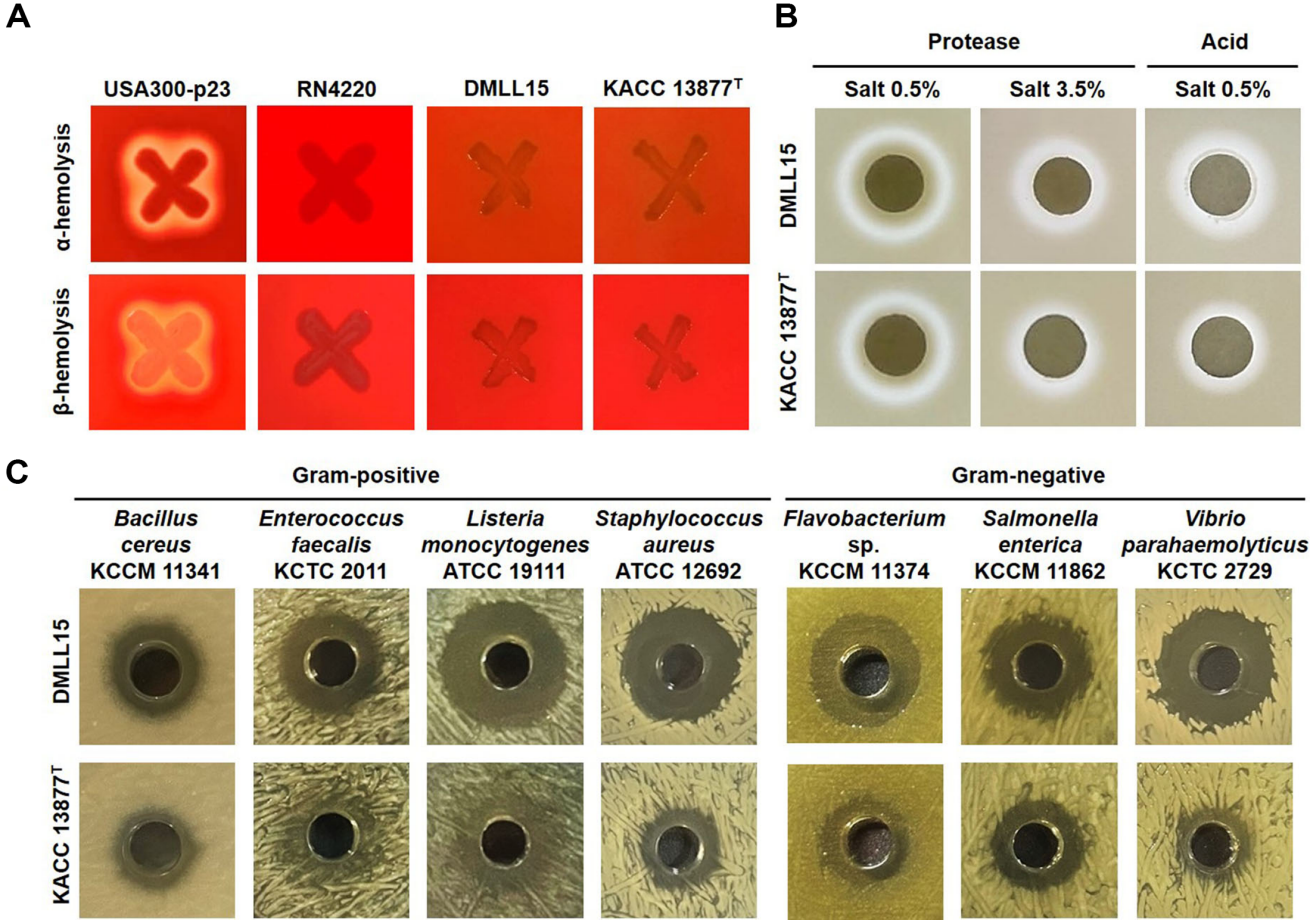
Hemolytic (A), enzyme (B), and antibacterial (C) activities of strain DMLL15. In A, *S. aureus* USA300-p23 and *S. aureus* RN4200 served as positive and negative controls for hemolytic activities, respectively. In B and C, type strain KACC 13877^T^ was used as the control for enzyme and antimicrobial activities. The formation of a clear zone around the filter paper disc and well is determined to be positive enzyme and antibacterial activity, respectively.

This experiment aimed to select candidate starter strains that are antibiotic-sensitive, possess strong enzyme activity, and exhibit robust antibacterial properties. Strains resistant to streptomycin and tetracycline were excluded from the candidate pool. Among the tested strains, nine exhibiting excellent protease activity at a 3.5%salt concentration were selected. Among the nine strains, *L. lactis* DMLL15, which exhibited the strongest antibacterial activity against all seven foodborne pathogens, was identified as the final candidate starter strain. *L. lactis* DMLL15 demonstrated sensitivity to nine antibiotics and did not exhibit hemolysis (Fig. 3A), confirming its safety as a potential starter culture. It also presented protease and acid production capacities (Figs. 3B and S1), as well as antibacterial activity against seven foodborne spoilage or pathogenic bacteria (Fig. 3C and Table S1). Ultimately, *L. lactis* DMLL15 strain holds promise for broader use in various fermented food systems, such as fermented dairy products, soybean fermentation, and kimchi fermentation. The proteolytic activity and acid production by the DMLL15 strain are anticipated to enhance the sensory properties of fermented foods, producing amino acids and organic acids through the decomposition of proteins and carbohydrates. Moreover, the remarkable antibacterial activity of *L. lactis* DMLL15 indicates its potential to improve the safety of fermented foods by inhibiting the growth of spoilage and pathogenic bacteria originating from raw materials or environmental sources during fermentation. Additionally, the antibacterial substances produced by *L. lactis* DMLL15 could be utilized not only in fermented foods but also as natural antimicrobial agents in food processing materials. These properties highlight its potential applications in extending the shelf life of minimally processed foods, improving the safety of ready-to-eat products, and even serving as a natural bio-preservative in food packaging systems.

## Supplemental Materials

Supplementary data for this paper are available on-line only at http://jmb.or.kr.



## Figures and Tables

**Table 1:** Oligonucleotides for identifying streptomycin and tetracycline resistance genes.

Antibiotics	Gene	Oligonucleotide sequence (5'-3')		Annealing temperature	Size (bp)	Reference
		Forward (5' → 3')	Reverse (5' → 3')			
Streptomycin	*strA*	CTTGGTGATAACGGCAATTC	CCAATCGCAGATAGAAGGC	55	500	[16]
	*strB*	ATCGTCAAGGGATTGAAACC	GGATCGTAGAACATATTGGC	56	500	[16]
	*aadA*	ATCCTTCGGCGCGATTTTG	GCAGCGCAATGACATTCTTG	56	282	[16]
	*aadE*	ATGGAATTATTCCCACCTGA	TCAAAACCCCTATTAAAGCC	50	565	[16]
Tetracycline	*tetK*	TTAGGTGAAGGGTTAGGTCC	GCAAACTCATTCCAGAAGCA	57	718	[17]
	*tetM*	ACAGAAAGCTTATTATATAAC	TGGCGTGTCTATGATGTTCAC	57	171	[18]

**Table 2:** Enzyme activities and acid production in *L. lactis* strains.

Strain	Protease		Acid
	Salt 0.5%*	Salt 3.5%	Salt 0.5%*
DMLL15	2.35 ± 0.07^de^	1.45 ± 0.07^e^	2.10 ± 0.14^fg^
AK5T01	2.05 ± 0.35^abcde^	0.40 ± 0.14^a^	1.85 ± 0.07^bcdefg^
AK5T02	2.25 ± 0.07^cde^	0.50 ± 0.00^a^	1.90 ± 0.14^bcdefg^
AK5T03	2.35 ± 0.07^de^	0.30 ± 0.00^a^	2.10 ± 0.14^fg^
AK5T04	2.25 ± 0.35^cde^	0.30 ± 0.14^a^	2.05 ± 0.07^efg^
AK5T05	2.35 ± 0.07^de^	0.30 ± 0.14^a^	1.85 ± 0.21^bcdefg^
AK5T06	2.40 ± 0.00^e^	0.50 ± 0.00^a^	1.70 ± 0.14^abcde^
AK0M06	2.25 ± 0.07^cde^	1.30 ± 0.14^cde^	1.70 ± 0.28^abcde^
AK0M07	2.30 ± 0.28^cde^	1.15 ± 0.07^bcd^	1.60 ± 0.14^abc^
AK0M08	2.30 ± 0.28^cde^	1.10 ± 0.14^bc^	1.85 ± 0.07^bcdefg^
AK0M09	2.20 ± 0.28^cde^	1.35 ± 0.21^de^	1.75 ± 0.21^bcdef^
AK0M10	2.45 ± 0.07^e^	1.40 ± 0.14^e^	1.40 ± 0.14^a^
AK0M11	2.35 ± 0.07^de^	0.50 ± 0.00^a^	1.85 ± 0.21^bcdefg^
AK0M12	2.25 ± 0.35^cde^	0.40 ± 0.14^a^	1.75 ± 0.07^bcdef^
AK0M13	2.35 ± 0.21^de^	0.50 ± 0.00^a^	1.60 ± 0.14^abc^
AK0M14	2.20 ± 0.14^cde^	0.30 ± 0.14^a^	1.55 ± 0.07^ab^
AK0M15	2.20 ± 0.42^cde^	0.40 ± 0.14^a^	1.90 ± 0.14^bcdefg^
AK0M16	1.85 ± 0.07^abc^	0.50 ± 0.00^a^	2.00 ± 0.00^defg^
AK0M17	2.25 ± 0.21^cde^	1.15 ± 0.21^bcd^	1.95 ± 0.07^cdefg^
AK0T02	1.65 ± 0.21^ab^	0.50 ± 0.00^a^	2.15 ± 0.07^g^
AK0T03	2.10 ± 0.14^bcde^	0.45 ± 0.07^a^	2.05 ± 0.07^efg^
AK0T04	1.90 ± 0.14^abcd^	0.30 ± 0.00^a^	2.05 ± 0.07^efg^
AK0T05	1.60 ± 0.28^a^	0.35 ± 0.21^a^	2.10 ± 0.14^fg^
AK0T06	1.90 ± 0.14^abcd^	0.50 ± 0.00^a^	2.00 ± 0.28^defg^
AK0T07	1.85 ± 0.07^abc^	0.40 ± 0.14^a^	2.10 ± 0.00^fg^
AK0T08	2.15 ± 0.07^cde^	0.50 ± 0.00^a^	1.75 ± 0.07^bcdef^
AK0T09	2.35 ± 0.07^de^	0.50 ± 0.00^a^	1.60 ± 0.14^abc^
AK0T10	2.30 ± 0.00^cde^	0.45 ± 0.07^a^	1.85 ± 0.21^bcdefg^
AK0T11	2.15 ± 0.21^cde^	0.50 ± 0.00^a^	1.65 ± 0.21^abcd^
AK0T12	2.05 ± 0.07^abcde^	0.25 ± 0.07^a^	1.90 ± 0.14^bcdefg^
AK0T13	2.30 ± 0.00^cde^	0.25 ± 0.07^a^	1.80 ± 0.14^bcdefg^
AK0T14	2.35 ± 0.21^de^	0.95 ± 0.07^b^	1.60 ± 0.14^abc^
AK0T19	2.40 ± 0.00^e^	1.10 ± 0.14^bc^	1.80 ± 0.00^bcdefg^
AK0T20	2.20 ± 0.14^cde^	1.05 ± 0.07^b^	1.95 ± 0.07^cdefg^

*The NaCl concentration in TSA is 0.5% (w/v).

Different superscripts in a column indicates significant difference at *p* < 0.05 by Duncan's multiple range test.
