# Supplementary material for: Potential Starter Candidate Based on Safety and Technological Evaluation of Lactococcus lactis from Kimchi, Korean Traditional Fermented Vegetables
Source: J Microbiol Biotechnol. 2025 Apr 24;35:e2501015. doi: 10.4014/jmb.2501.01015 (PMC12089941; doi:10.4014/jmb.2501.01015)
Supplement: Supplementary file 1 [file jmb-35-e2501015-supple.pdf]

J. Microbiol. Biotechnol. 2025. 35: e2501015
https://doi.org/10.4014/jmb.2501.01015

Supplementary Figure and Table

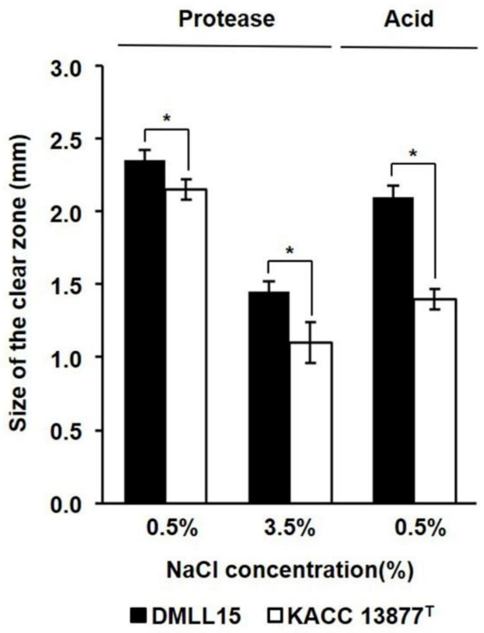

Fig. S1. Qualitative enzyme activity and acid production measure in clear zone around filter paper disc containing DMLL15. The mean values of replicates are presented. Asterisks (*) indicate significant difference at $p < 0.05$ by an independent t-test.

Table S1. Qualitative antibacterial activity measure in clear zone around well containing DMLL15.

	DMLL15	KACC 13877^T
Gram-positive		
Bacillus cereus KCCM 11341	1.55 ± 0.07^b	1.10 ± 0.14^a
Enterococcus faecalis KCTC 2011	2.20 ± 0.14^b	0.90 ± 0.14^a
Listeria monocytogenes ATCC 19111	3.85 ± 0.07^b	1.05 ± 0.07^a
Staphylococcus aureus ATCC 12692	3.65 ± 0.07^b	1.25 ± 0.07^a
Gram-negative		
Flavobacterium sp. KCCM 11374	2.85 ± 0.07^b	1.45 ± 0.07^a
Salmonella enterica KCCM 11862	2.65 ± 0.07^b	1.70 ± 0.14^a
Vibrio parahaemolyticus KCTC 2729	3.60 ± 0.14^b	1.05 ± 0.21^a

Different superscripts in a row indicate significant difference at $p < 0.05$ by an independent t-test.
